# Different Patterns of Ecological Divergence Between Two Tetraploids and Their Diploid Counterpart in a Parapatric Linear Coastal Distribution Polyploid Complex

**DOI:** 10.3389/fpls.2020.00315

**Published:** 2020-03-19

**Authors:** Mariana Castro, João Loureiro, Albano Figueiredo, Miguel Serrano, Brian C. Husband, Sílvia Castro

**Affiliations:** ^1^Centre for Functional Ecology, Department of Life Sciences, University of Coimbra, Coimbra, Portugal; ^2^Centre for Studies in Geography and Spatial Planning, Department of Geography and Tourism, Faculty of Arts and Humanities, University of Coimbra, Coimbra, Portugal; ^3^Department of Botany, Faculty of Pharmacy, University of Santiago de Compostela, Santiago de Compostela, Spain; ^4^Department of Integrative Biology, University of Guelph, Guelph, ON, Canada

**Keywords:** parapatric distribution, cryptic diversity, diploids, *Jasione maritima*, niche modeling, tetraploids

## Abstract

Polyploidization is a widespread mechanism of evolutionary divergence in flowering plants. Ecological divergence of polyploid lineages has been proposed as a key process shaping the distribution of cytotypes in nature (niche shift hypothesis); however, evidence for the role of niche separation in replicated diploid-polyploid species pairs is still needed. This study aimed to assess the role of abiotic factors shaping current cytotype distributions. For that, we examined the distribution and environmental niches of two varieties recognized in diploid-tetraploid *Jasione maritima* across the species range and within a putative contact zone on the Iberian Peninsula. We counted chromosomes, screened for ploidy across Iberian Peninsula and characterized environmental requirements using niche modeling tools. We found that *J. maritima* var. *maritima* is composed by diploids with disjunct distribution in the west coast of France and northwest Iberian Peninsula, and by tetraploids in Iberian Peninsula, while var. *sabularia* is tetraploid. In the Iberian Peninsula, two parapatric contact zones along a linear coastal distribution were detected, one between diploid and tetraploid var. *maritima*, and the other between tetraploids of the two varieties. Environmental variables of diploid populations from France are distinct from those of southern diploid populations, which are more similar to tetraploids. In general, niche modeling results are congruent with the observed distribution patterns, although the results suggest a wider contact zone between varieties and cytotypes. Tetraploids of both varieties revealed different degrees of environmental divergence in comparison with their diploid counterpart. Tetraploid var. *sabularia* differed environmentally from diploids suggesting niche divergence. In contrast, tetraploid var. *maritima* overlapped with diploid environmental niche and currently occupies its entire predicted range, whereas diploids are restricted to northern areas of their suitable environment. Differences in ecological envelopes facilitate the recognition of functional units of biodiversity within polyploid groups, allowing the study of factors related to post-polyploidization divergence. Thus, whereas changes in environmental requirements may have allowed tetraploid var. *sabularia* to spread in habitats not favorable to diploids, other factors are involved with the distribution of diploid and tetraploid var. *maritima*.

## Introduction

Whole genome duplication leading to polyploidy is a widespread mechanism of plant evolution and diversification (Soltis and Soltis, 1999; Soltis et al., 2010; Jiao et al., 2011). Estimates of the incidence of polyploidy in angiosperms range from 20 to 40% (e.g., Stebbins, 1938, 1950; Wood et al., 2009) and is considerably higher in specific geographical regions (e.g., 69–87% in the Arctic Flora, Brochmann et al., 2004; 37–49% in the Mediterranean Basin, Marques et al., 2017). Most studies of the incidence of polyploidy are based on chromosome counts obtained during taxonomic studies; however, due to technical and logistical difficulties, such studies are usually based on a few counts per species, which can limit one’s ability to detect multiple cytotypes (Bennett, 1998; Soltis et al., 2007; Marques et al., 2017). With the emergence of high throughput methods such as flow cytometry, the number of studies focused on the incidence of polyploidy has increased (Kron et al., 2007; Husband et al., 2013) and revealed that the extent of polyploidy is underestimated in many plant species (Marques et al., 2017).

Flow cytometry has enabled detailed studies of polyploid complexes and has thus provided novel insights into geographic patterns of cytotype diversity, from within populations to across the entire species geographic range (e.g., *Aster amellus*, Castro et al., 2012; *Chamerion angustifolium*, Husband and Schemske, 1998; *Erysimum mediohispanicum*, Muñoz-Pajares et al., 2018; *Knautia arvensis* agg., Kolář et al., 2009; *Mercurialis annua*, Buggs and Pannell, 2007; *Ranunculus adoneus*, Baack, 2004). These large-scale studies of diploid-polyploid complexes have revealed a wide variety of cytotype compositions and geographic distributions, confirming that polyploidy is a complex and dynamic process in nature. According to Petit et al. (1999), the majority of cytotypes in polyploid complexes grow in close proximity, at least in part of their distribution range, and form parapatric or sympatric contact zones (e.g., Kolář et al., 2009; Castro et al., 2012; Muñoz-Pajares et al., 2018). Cytotype distributions are the result of multiple processes, including the evolutionary history of the complex, frequency of polyploid formation, divergent abiotic tolerances and biotic interactions (e.g., competition, herbivores), as well as dispersal abilities and inter-cytotype breeding barriers (Petit et al., 1999; Levin, 2002; Lexer and van Loo, 2006). While cytotypes in most polyploids have complex distributions, it is rarely known which process or combination of processes account for such complexity.

Abiotic factors, such as temperature and precipitation, play a key role in shaping distributions of species and biodiversity. Because polyploidization creates novelty, it may drive direct or indirect changes in phenotypes and result in shifts in ecological tolerances (Levin, 1975; Husband and Schemske, 2000; Maherali et al., 2009; Ramsey, 2011; Manzaneda et al., 2012, 2015; Hao et al., 2013). One of the immediate effects of polyploidization is the increase in cell size (Stebbins, 1971; Masterson, 1994), which is related to changes in physiological traits. For example, genome duplication frequently leads to increased stomata size and lower stomata densities (Li et al., 1996; Maherali et al., 2009), thicker epidermis (Li et al., 1996), or changes in xylem structure, cell size and wall thickness, and lignin content (Nassar et al., 2008; Maherali et al., 2009; Hao et al., 2013). These changes can affect gas exchange and water transport and generate trade-offs between water transport efficiency and cavitation risk, and often leads to greater drought tolerance in polyploids than their diploid relatives (e.g., Maherali et al., 2009; Manzaneda et al., 2012, 2015; Hao et al., 2013). Such physiological differences acquired by polyploids may have significant ecological implications (Ramsey and Schemske, 2002), allowing ecological niche expansion of the polyploid lineages (niche shift hypothesis; Levin, 1975; Fowler and Levin, 1984; Husband and Schemske, 2000; Parisod and Broennimann, 2016). Niche differentiation between polyploids and their diploid relatives may thus enable polyploids to expand to areas unoccupied by diploids and avoid direct competition (Levin, 1975, 2003; Husband and Schemske, 2000); however, evidence for the role of environment in accounting for distribution differences between diploids and polyploids remains largely unexplored.

Niche modeling (e.g., Ecological Niche Modeling; Warren et al., 2008, 2010) and multivariate analyses of predictor variables (Broennimann et al., 2012) are useful tools for examining relationships between large-scale cytotype distributions and environmental tolerances. These methods take advantage of global climate and habitat databases to relate the geographic distributions of diploid and polyploid populations to spatial environmental data, supporting the creation of predictions about niche shifts or niche conservation associated with whole genome duplication (e.g., Glennon et al., 2012; Godsoe et al., 2013; Thompson et al., 2014; Visger et al., 2016; Muñoz-Pajares et al., 2018; López-Jurado et al., 2019; Molina-Henao and Hopkins, 2019). Such approaches are powerful in scope and useful for generating hypotheses that can be further tested using manipulative experiments (Glennon et al., 2014; Marchant et al., 2016), such as reciprocal transplants (e.g., *C. angustifolium*, Martin and Husband, 2013).

The genus *Jasione* L. (Campanulaceae) comprises 16 species found mostly in the Mediterranean region (Pérez-Espona et al., 2005). It is a relatively old genus (Bokhari and Sales, 2001; Haberle et al., 2009) and diversification appears to have occurred recently, possibly during the last glaciation cycle in Europe (Bokhari and Sales, 2001; Pérez-Espona et al., 2005). The Iberian Peninsula is the center of morphological diversity in the genus, with variation among the 10 accepted species (Sales and Hedge, 2001) in life history, ploidy and habitat (Bokhari and Sales, 2001; Sales and Hedge, 2001, Rubido-Bará et al., 2010). *Jasione maritima* (Duby) Merino comprises two varieties: *J. maritima* var. *maritima* occurring in western France and in northwestern Spain, and *J. maritima* var. *sabularia* (Cout.) Sales and Hedge from the north coast of Portugal (Sales and Hedge, 2001). The species also has been cited for one locality in the coast of the Basque Country, near Bilbao (Guinea, 1949), but it has been considered doubtful and the species has not been located again in this region (Aseginolaza et al., 1984). The var. *maritima* in France was identified as diploid (2*n* = 2*x* = 12 chromosomes; Delay, 1967), whereas plants in Spain are tetraploid (2*n* = 4*x* = 24 chromosomes; Lago Canzobre and Castroviejo, 1992; Rubido-Bará et al., 2010); var. *sabularia* has been described as strictly tetraploid (Leitão and Paiva, 1988). However, a recent study reported the presence of two genome size categories in some Galician populations of var. *maritima*, namely 2C = 3.44 ± 0.04 pg in the northern locations and 2C = 6.62 ± 0.23 pg in the southern ones (Rubido-Bará et al., 2010). This may suggest the presence of ploidy variation on the Iberian Peninsula. The distribution of diploids in northern locations and tetraploids in southern ones may suggest niche differentiation between cytotypes, making this system ideal to explore the role of environmental variables in niche separation.

In this study we explored the role of niche divergence driving current cytotype and varieties distribution patterns in *J. maritima* complex. For that, we (1) investigate the diversity of cytotypes throughout the range of *J. maritima*, (2) describe the frequency and distribution of cytotypes within and among natural populations, and (3) examine the correspondence between environmental variables and cytogeographic patterns in the species. We quantified ploidy variation using chromosome counts and flow cytometry over the species’ distribution in the Iberian Peninsula, a putative contact zone between the reported cytotypes (from northwestern to central coast). We then used niche modeling tools and multivariate analyses to determine whether the observed cytotype distribution patterns could be explained by environmental variables. The environmental analyses were performed at two spatial scales: (1) the entire distribution range (1 km resolution) and (2) the northwestern coast of the Iberian Peninsula (100 m resolution). We tested the hypothesis that cytotype distribution in *J. maritima* reflects the ecological divergence between diploids and their polyploid descendants.

## Materials and Methods

### Study System and Field Sampling

*Jasione maritima* (Duby) Merino is a perennial herb that grows in sand dune systems from Ferrol (Galicia, Spain) to São Jacinto (Aveiro, Portugal) in the Iberian Peninsula, and in the west coast of France (Sales and Hedge, 2001). The plant forms a leafy rosette in winter and produces capituliform inflorescences in the summer. The inflorescence is composed of blue to lilac, insect pollinated flowers, each producing dozens of small seeds that germinate from autumn to late winter (Sales and Hedge, 2001; M. Castro, field observations).

Field surveys were conducted during the flowering season (June and July), from 2013 to 2015, within and beyond the known distribution limits of *J. maritima* in the Iberian Peninsula. All locations visited were geo-referenced (see Supplementary Table 1 for the 35 *J. maritima* populations). Plants in each population were identified based on indumentum and leaf shape following Flora Iberica (Sales and Hedge, 2001) as *J. maritima* var. *maritima* or *J. maritima* var. *sabularia*. Within each population, we randomly sampled 5–6 fresh leaves from each of 4–30 individuals and stored them in hermetic plastic bags at 4–8°C to assess genome size and DNA ploidy using flow cytometry. For chromosome counts, seeds from up to 15 plants were collected in paper bags from selected locations, based on preliminary genome size estimates and including one population of each genome size category (Supplementary Table 1). Herbarium specimens were collected, and vouchers deposited in SANT herbarium (Supplementary Table 1).

### Chromosome Counts

Chromosome counts were attained following the protocol of Goldblatt and Takei (1993) with some modifications. Briefly, seeds from one population per genome size category (Supplementary Table 1) were germinated and grown in 1L pots with commercial soil in an experimental garden. Actively growing root tips were harvested and pre-treated in 0.002 M aqueous 8-hydroxyquinoline at room temperature for 4 h and 30 min, after which root tips were fixed in a solution of 3:1 of 96% ethanol and glacial acetic acid for at least 24 h at 4°C. Roots tips were then hydrolyzed in 1 N HCl at 60°C in a sand bath for 5 min, submerged in Schiff reagent (based in Greilhuber and Ebert, 1994) for 1 h and 30 min, washed in Sulfur water three times for 10 min periods, and finally squashed under a glass cover in aseptic orcein 2%. Chromosome spreads were observed using a Nikon Eclipse 80i light microscope and photographed using a Nikon Plan Apo VC 100 × /1.40 oil-immersion lens, with a Q Imaging Retiga 2000R Fast 1394 digital camera and Q-Capture Pro v.7 software. Based on the 2*n* chromosome counts, plants were assigned to a ploidy level (diploid for 2*n* = 12, or tetraploid for 2*n* = 24). We used these same plants as reference material to determine DNA ploidy of each genome size category and estimate the DNA ploidy of the remaining individuals using flow cytometry.

### Genome Size and DNA Ploidy Estimates

We measured genome size and DNA ploidy in all plants using flow cytometry. Following Galbraith et al. (1983), 50 mg of leaf material of each sample was chopped with 50 mg of leaves of an internal reference standard (*Solanum lycopersicum* “Stupické”, hereafter S.l., with 2C = 1.96 pg; Doležel et al., 1992, 2007) using a sharp razor blade in a glass Petri dish with 1 ml of WPB buffer (0.2 M Tris–HCl, 4 mM MgCl_2_.6H_2_O, 1% Triton X-100, 2 mM EDTA Na_2_.2H_2_O, 86 mM NaCl, 10 mM metabisulfite, 1% PVP-10, pH adjusted to 7.5 and stored at 4–8°C; Loureiro et al., 2007). The nuclear suspension was filtered through a 50 μm nylon filter to remove coarse debris and 50 μg ml^–1^ propidium iodide (PI; Fluka, Buchs, Switzerland) and 50 μg ml^–1^ RNAse (Fluka) were added to stain the DNA and avoid staining dsRNA, respectively. After 5 min of incubation, the samples were analyzed in a Partec CyFlow Space flow cytometer (532 nm green solid-state laser, operating at 30 mW; Partec GmbH., Görlitz, Germany). The results were visualized using Partec FloMax software v2.4d (Partec GmbH, Münster, Germany) in four graphics: histogram of fluorescence pulse integral in linear scale (FL); forward light scatter (FS) vs. side light scatter (SS), both in logarithmic (log) scale; FL vs. time; and FL vs. SS in log scale. To remove debris, a polygonal region was defined in the FL vs. SS histogram and subsequently applied to all graphics. At least 1300 nuclei from sample and standard G_1_ peaks were analyzed per sample (Suda et al., 2007). Only samples with a coefficient of variation (CV) of 2C peak <5% were included; when a sample failed to meet this standard, it was re-prepared and analyzed until such quality was achieved (Greilhuber et al., 2007). In 15 populations (six diploid var. *maritima*, six tetraploid var. *maritima* and three tetraploid var. *sabularia*), 2–10 individuals were analyzed individually for genome size. For the remaining individuals and populations, the samples were pooled (5–6 individuals plus the reference standard) providing estimates of DNA ploidy only. The holoploid genome size (2C in pg; *sensu* Greilhuber et al., 2005) was calculated using the formula:

$$J.m\,a\,r\,i\,t\,i\,m\,a\,2\,C\,\text{nuclear}\,\text{DNA}\,\text{content}\,\left(\mathrm{pg}\right)\;=\frac{J.m\,a\,r\,i\,t\,i\,m\,a\,\mathrm{G1}\,\text{peak}\,\text{mean}}{S.l.\mathrm{G1}\,\text{peak}\,\text{mean}}\times S.l.\text{genome}\,\text{size}$$

DNA ploidy was inferred for each sample based on the chromosome counts and genome size estimates obtained for the selected populations. The monoploid genome size (1Cx; *sensu* Greilhuber et al., 2005; mass in pg) was calculated by dividing the holoploid genome size (2C) by the assigned DNA ploidy. Based on plant identification and ploidy level estimates, each population was classified as follows: diploid *J. maritima* var. *maritima* (2*x* var. *maritima*), tetraploid *J. maritima* var. *maritima* (4*x* var. *maritima*) or tetraploid *J. maritima* var. *sabularia* (4*x* var. *sabularia*).

Descriptive statistics of 2C genome size were calculated for each cytotype (mean, SD, CV, and range) based on individual flow cytometric estimates, only. Mean and SD were also calculated for the 1Cx genome size. To assess differences between diploids and tetraploids in 2C and 1Cx genome sizes, we used generalized linear models (GLM) (Bolker et al., 2009) with a Gaussian distribution and an identity link function. Cytotype was treated as a fixed factor and genome size as the response variable. Statistical analyses were performed in R software version 3.0.1 (R Core Development Team, 2016), using the packages “car” for Type-III analysis of variance (Fox et al., 2015), “lme4” for generalized linear models (GLM; Bates et al., 2014) and “multcomp” for multiple comparisons after Type-III analysis of variance (Hothorn et al., 2017).

### Environmental Niche Modeling

Environmental niches of the two varieties of *J. maritima* and the cytotypes within var. *maritima* (i.e., 2*x* var. *maritima*, 4*x* var. *maritima* and 4*x* var. *sabularia*) were compared using GLM analyses and niche modeling tools. We conducted these analyses at two spatial scales: (1) entire geographic distribution of *J. maritima* (in Portugal, Spain, and France); and (2) the putative contact zone (in the northwest Iberian Peninsula).

The presence database was generated based on our field observations in the Iberian Peninsula, herbarium specimens from COI and SANT (acronyms following Thiers, 2017), literature survey of karyologic reports with geographical information, and GBIF database.^1^ For France, only karyologic reports and GBIF information were used. All reports from France were classified as diploid, based on previous chromosome counts (Delay, 1967). The final database included 40 records for diploid var. *maritima*, 21 records for tetraploid var. *maritima* and 18 records for tetraploid var. *sabularia*.

To examine environmental niches at the scale of the entire species range, 19 bioclimatic variables (Bio1-Bio19) plus elevation were extracted for all species occurrences from the Worldclim database^2^ at a resolution of 30 arc-seconds (approx. 1 km; Supplementary Table 2), using R package “dismo” (Hijmans et al., 2017). At the scale of the putative contact zone, the following set of variables were used at 100 m resolution to account for the restricted habitat of the species: elevation;^3^ topographic variables including aspect (in degrees: 310–360° and 0–45° exposed to N – cooler; 45–135° exposed to E; 135–220° exposed to S – hotter; 220–310° exposed to W – humid), slope, topographic position index (TPI; mm) and incoming solar radiation for the summer period;^4^ climatic variables including summer mean temperature (June to August) and mean annual precipitation;^5^ lithology;^6^ and distance from the sea (Supplementary Table 3). Additionally, latitude and longitude were also included in both approaches (Supplementary Tables 2, 3). Different variables were used in the two approaches because of differences in the available parameters at the two resolution scales.

**FIGURE 1:**
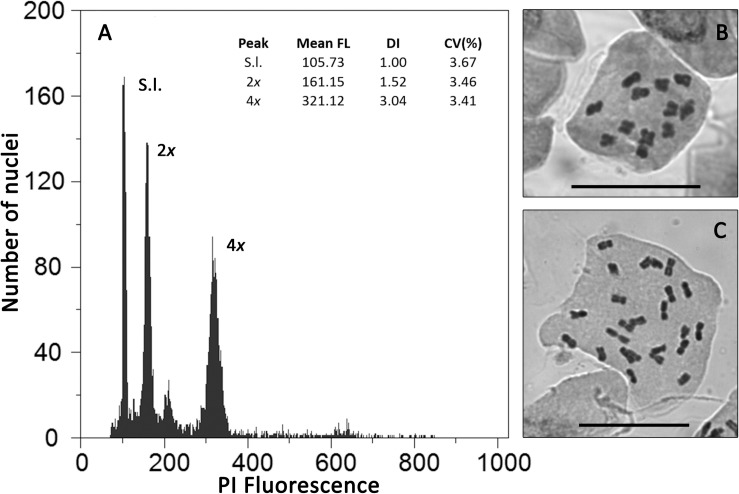
Cytogenetic diversity in *Jasione maritima*: **(A)** Flow cytometric histogram of relative propidium iodide fluorescence intensity (PI Fluorescence) of nuclei isolated from fresh leaves of *S. lycopersicum* “Stupické” (S.l.; reference standard with 2C = 1.96 pg) and of *Jasione maritima* diploid (2*x*) and tetraploid (4*x*) individuals; **(B)** Chromosome plate of a diploid *J. maritima* var. *maritima* individual from population MS003 (2*n* = 2*x* = 12 chromosomes; bar = 20 μm, Table 1); **(C)** Chromosome plate of a tetraploid *J. maritima* var. *maritima* individual from population SC116 (2*n* = 4*x* = 24 chromosomes; bar = 20 μm, Table 1). In A, for each peak, the mean relative fluorescence (Mean FL), DNA index (DI, Mean FL of *J. maritima* peak/Mean FL of the reference standard) and coefficient of variation of the peak (CV, in%) are provided.

Differences in extracted variables among cytotypes were evaluated using GLMs, with cytotype as a fixed factor and each variable as a response variable. A Gaussian distribution with an identity link function was used for continuous variables and a Poisson distribution with a log link function was used for discrete variables. Exploratory principal component analyses (PCA) were also performed to evaluate the contribution of each variable for the total variance. Correlations between the variables were obtained using Pearson/Spearman coefficients. For each set of variables with correlation values higher than 0.7, only one was selected. Taking into account the results of the correlations and the PCAs (Supplementary Tables 2–4 and Figure 1), a set of non-correlated variables were selected for niche modeling analyses: in the entire range approach, mean diurnal range, isothermality, temperature seasonality, precipitation of the wettest month and precipitation seasonality (Supplementary Tables 2, 4) were selected; for the putative contact zone approach, slope, summer mean temperature, mean annual precipitation and distance to coast (Supplementary Tables 3, 4) were used.

Niche modeling analyses were performed with maximum entropy modeling (MaxEnt) using the R software package “biomod2” (Thuiller et al., 2016). Spatial predictive models were calibrated based on the selected variables and on presence/absence data. Absences were derived from 19 confirmed observations from field surveys, occurrence records for the other two cytotypes (namely, in the 2*x* var. *maritima* dataset, 4*x* var. *maritima* and 4*x* var. *sabularia* populations were considered as absences, and vice-versa in the 4*x* var. *maritima* and 4*x* var. *sabularia* datasets) and 5000 pseudo-absences. For pseudo-absences, a buffer around each presence point (of 3 km in the entire range approach and 300 m in the contact zone approach) was applied and 5000 points were randomly selected within the remaining area (defined as background points). To reduce uncertainty and to produce robust models, each technique was replicated 30 times using random subsets obtained from each dataset. The presence database of each entity was divided randomly into training (70%) and test (30%) subsets (Phillips et al., 2006; Araújo and New, 2007). All subsets were statistically independent, since in each subset, each occurrence was used only once, as training or as test occurrence (Phillips, 2008). Models were evaluated based on the independent accuracy measure AUC of ROC (area under the curve of the receiver operating characteristic), and only models with AUC > 0.7 were combined to produce the final model for each entity (Supplementary Table 5). The final models were converted to a binary format (using the default threshold of 0.5) and were used to calculate niche overlap between the entities.

The percentage of niche overlap was quantified for the three paired cytotype comparisons (i.e., 2*x* var. *maritima* vs. 4*x* var. *maritima*; 2*x* var. *maritima* vs. 4*x* var. *sabularia*; and 4*x* var. *maritima* vs. 4*x* var. *sabularia*) at the whole range and putative contact zone scales. Niches were compared through an ordination approach using a PCA calibrated with environmental values (Di Cola et al., 2017) using “ecospat” (Broennimann et al., 2012) and “raster” (Hijmans et al., 2017) R packages. The PCA calculates the occurrence density and environmental factor density along environmental (principal component) axes for each pixel, maximizing the ecological variance of the areas of the cytotypes. Then, PCA scores of the cytotype distributions were projected onto a grid of cells bounded by the maximum and minimum PCA scores; this allows the visual assessment of the overlap and dynamics of the environmental niches of the cytotypes being compared (Di Cola et al., 2017). All models and analyses were performed in the R 3.3.0 environment (R Core Development Team, 2016).

## Results

### Cytogenetic Diversity

Flow cytometric histograms were of good quality, with all samples used to assess genome size having CV values below 5% (Figure 1A and Supplementary Table 6). Each genome size category corresponded to different chromosome numbers (Figure 1 and Table 1): individuals with 12 chromosomes presented average genome sizes of 2.98 pg/2C (range: 2.84 – 3.10 pg/2C; Figures 1A,B), while individuals with 24 chromosomes had average genome sizes of 6.06 pg (range: 5.80–6.36 pg; Figures 1A,C), corresponding to diploid and tetraploid cytotypes, respectively.

**TABLE 1:** Chromosome number and genome size variation in *Jasione maritima*.

Taxon	Ploidy level	Chr. no.	Holoploid G.s. (2C, pg)					Monoploid G.s. (1C*x*, pg)		*N*_ind_	*Npop*
				
			Mean	SD	CV (%)	Min	Max	Mean	SD		
*J. maritima* var. *maritima*	2*x*	12	2.98^a^	0.07	2.4%	2.84	3.10	0.25^n.s.^	0.01	24	6
	4*x*	24	6.06^b^	0.11	1.9%	5.80	6.36	0.25^n.s.^	0.01	38	6
*J. maritima* var. *sabularia*	4*x*	24	5.99^b^	0.18	1.2%	5.76	6.36	0.25^n.s.^	0.01	22	3

*DNA ploidy levels, chromosome number (2n, Chr. no.), holoploid (2C) and monoploid (1Cx) genome size are presented in picograms (pg). For genome size estimates, mean, standard deviation (SD), coefficient of variation (CV,%), minimum and maximum values are given. Different letter corresponds to statistically significant differences at *P* < 0.05; n.s. denotes no significant differences at *P* > 0.05. The columns *N*_*ind*_ and *N*_*pop*_ provide the number of analyzed individuals and populations, respectively.*

*Jasione maritima* var. *maritima* presented both diploid and tetraploid individuals, while var. *sabularia* was tetraploid, only. Significant differences were observed in holoploid genome sizes (*F*_2_,_81_ = 5020, *P* < 0.001), with differences occurring between cytotypes (*P* < 0.05), but not between varieties within the tetraploid cytotype (*P* > 0.05; Table 1). Thus, tetraploids from the two varieties can only be distinguished based on morphologic traits. No statistically significant differences were observed between varieties and cytotypes regarding monoploid genome size (*F*_2_,_81_ = 0.534, *P* = 0.588; Table 1; Supplementary Table 6).

### Cytotype Distributions in the Iberian Peninsula

**FIGURE 2:**
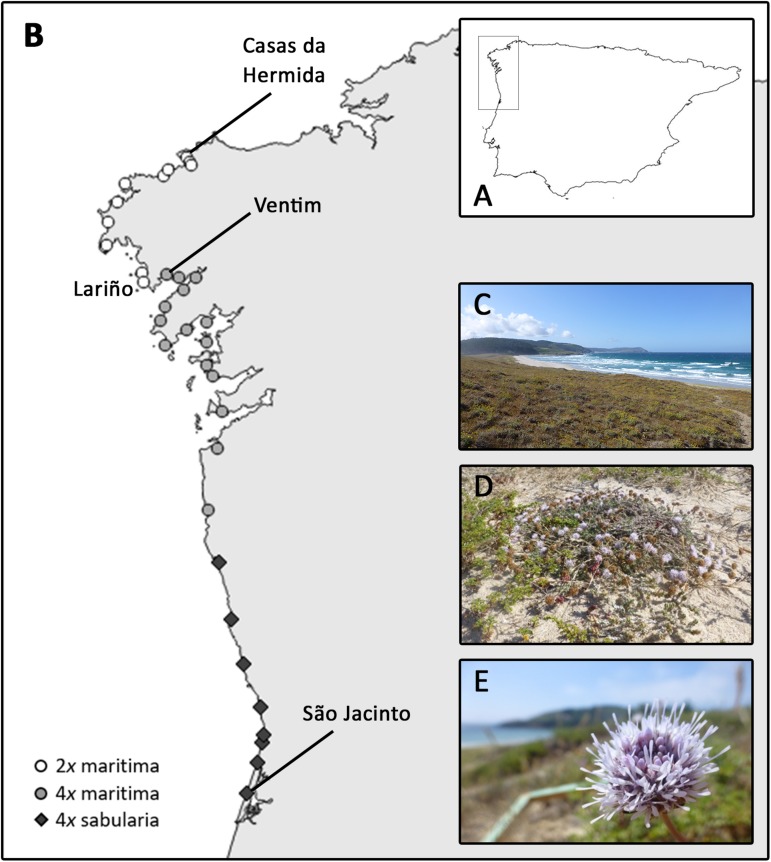
*Jasione maritima* large-scale cytotype distribution based on flow cytometric analyses: **(A)** Iberian Peninsula with the screening area highlighted; **(B)** cytotype distribution in the surveyed area; **(C)** dune habitat, **(D)** habit, and **(E)** inflorescence of diploid *J. maritima* var. *maritima* of population SC150 (Table 1). Circles represent populations of *J. maritima* var. *maritima*, white correspond to diploids and light gray correspond to tetraploid populations, and dark gray diamonds refer to tetraploid *J. maritima* var. *sabularia* populations.

We assessed the DNA ploidy of 986 individuals from 35 natural populations spanning the distribution range of *J. maritima* in the Iberian Peninsula (Figure 2; Supplementary Table 1). The large-scale survey revealed diploid and tetraploid single-ploidy populations. As described above, *J. maritima* var. *maritima* comprises both diploids and tetraploids, while *J. maritima* var. *sabularia* is only tetraploid. The taxa and cytotypes are distributed parapatrically; diploid var. *maritima* occurs in the north, from Casas da Hermida to Lariño (Spain), tetraploid var. *maritima* occurs from Ventim (few kms south of the southern-most diploid population) to the south along the northwestern Spanish coast; and tetraploid var. *sabularia* occurs along the northern coast of Portugal (Figure 2). In the Iberian Peninsula, tetraploids occupy a wider area than diploids (Figure 2).

### Environmental Niche

Univariate analyses of environmental variables for the entire distribution of *J. maritima* revealed significant differences for all variables except elevation (see Supplementary Table 2 for *F* and *P* values). Diploid var. *maritima* tends to occur in areas with lower temperatures and higher precipitation levels in the driest period (i.e., in summer) than tetraploid var. *maritima* and tetraploid var. *sabularia*, which both colonize warmer areas with drier summers (Supplementary Table 2). In particular, when compared with both tetraploid varieties, the diploid var. *maritima* occurs in areas with significantly lower annual mean temperature, mean temperatures of quarter periods, precipitation seasonality and annual precipitation, and although precipitation was significantly lower in wetter and colder periods, it was significantly higher in the driest and warmest periods (all *P* < 0.05; Supplementary Table 2). Tetraploid var. *maritima* occurs in climatic conditions that are, in general, more similar to the tetraploid var. *sabularia* than to diploids (Supplementary Table 2), namely for annual mean temperature, mean temperatures of quarter periods and precipitation of the wettest quarter (*P* > 0.05; Supplementary Table 2), not differing from diploid for isothermality and maximum temperature of the warmest month (*P* > 0.05; Supplementary Table 2). Interestingly, the areas occupied by the tetraploid var. *maritima* presented intermediate and significantly different values for precipitation variables (*P* < 0.05; except for precipitation in the wettest month and quarter, *P* > 0.05; Supplementary Table 2). The tetraploid var. *sabularia* occurs in areas with significantly higher precipitation seasonality, reflected in significantly higher precipitation in the coldest quarter, while having significantly lower precipitation in driest and warmest periods in comparison with both cytotypes of var. *maritima* (all *P* < 0.05; Supplementary Table 2). The PCA revealed two clusters of diploid populations; the French populations were clearly separated from the Iberian populations along axis 1 (high loadings for example for precipitation and temperature seasonality, Supplementary Figure 1A and Supplementary Table 4). In contrast, the diploid Iberian populations cluster near the tetraploid populations of both varieties (Supplementary Table 2 and Supplementary Figure 1A).

When analyzed at finer spatial resolution (100 m) in the Iberian Peninsula (the putative contact zone), significant differences among varieties and cytotypes were obtained for all variables except elevation, aspect and distance from the sea (see Supplementary Table 3 for *F* and *P* values). Diploid var. *maritima* colonizes areas with slightly higher elevation and facing west (although no significant differences were observed between cytotypes and varieties), and with significantly steeper slopes, lower topographic index, higher mean annual precipitation and lower mean summer temperatures (cooler areas) than tetraploid var. *sabularia* (all *P* < 0.05; Supplementary Table 3). The southern areas colonized by tetraploid var. *sabularia* are thus marked by significantly lower slopes (*P* < 0.05) facing south-southwest and by significantly hotter and drier environments than those occupied by diploids (i.e., significantly higher mean summer temperature and mean annual precipitation, *P* < 0.05; Supplementary Table 3). Tetraploid var. *maritima* tends to occur in areas environmentally similar to the diploids (i.e., similar mean summer temperatures and mean annual precipitation; *P* > 0.05), with the exception of slope values and topographic indexes, which were different from diploids (*P* < 0.05) and similar to those obtained in tetraploid var. *sabularia* localities (*P* > 0.05; Supplementary Table 3). Slope and topographic index suggest that the diploid var. *maritima* is usually located at interdunes depressions (low negative TPI and steeper slopes), while the tetraploids of both varieties usually grow in flat areas (TPI close to zero and low slopes). Consequently, in the PCA, the two cytotypes of var. *maritima* are clustered together and are separated from tetraploid var. *sabularia* along axes 1 and 2, which are associated with mean annual precipitation and mean summer temperature (Supplementary Tables 3, 4 and Supplementary Figure 1B).

**FIGURE 3:**
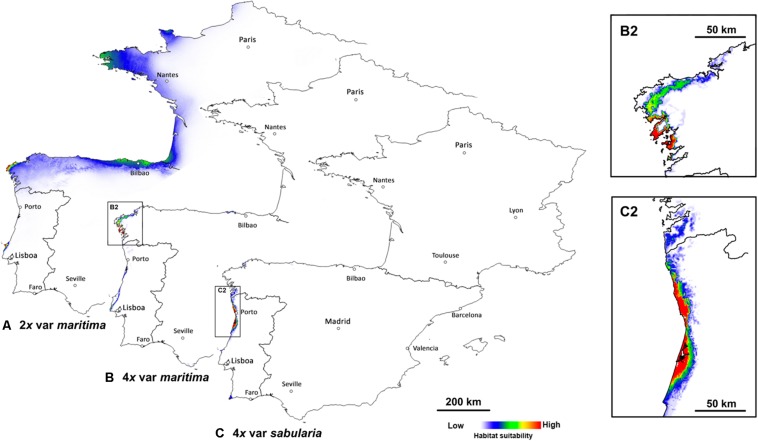
Predicted geographic niches for **(A)** diploids of *Jasione maritima* var. *maritima* (2*x* var. *maritima*), **(B)** tetraploids of *J. maritima* var. *maritima* (4*x* var. *maritima*), plus an inset of the area with higher probability of occurrence (B2), and **(C)** tetraploids of *J. maritima* var. *sabularia* (4*x* var. *sabularia*), plus an inset of the area with higher probability of occurrence (C2), considering the entire distribution area. White to red color gradient represent habitats with higher and lower probability of occurrence of the cytotype, respectively.

**FIGURE 4:**
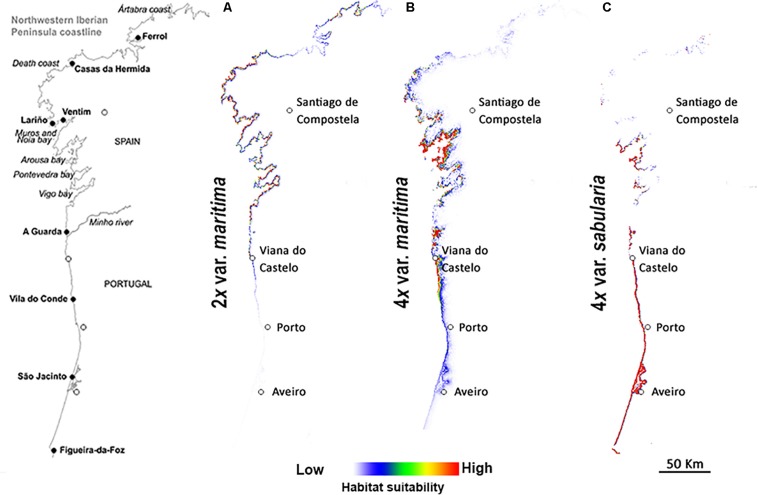
Predicted geographic niches for **(A)** diploids of *Jasione maritima* var. *maritima*, **(B)** tetraploids of *Jasione maritima* var. *maritima*, and **(C)** tetraploids of *Jasione maritima* var. *sabularia*, in the sympatric zones at the Iberian Peninsula. White to red color gradient represent habitats with higher and lower probability of occurrence of the cytotype, respectively.

Predicted distributions based on niche modeling confirm the parapatric distributions of the three cytotypes (Figures 3, 4). Across the entire geographic distribution of *J. maritima*, models showed high habitat suitability for diploid var. *maritima* (Figure 3A) on the northwest coast of the Iberian Peninsula, the Death Coast of Spain where Iberian populations are currently located (Figure 2), in the south and northwest coast of France where the plant currently occurs, and on the coast near Bilbao, where no natural populations currently occur (Figure 3A). By contrast, the suitable habitats for tetraploids are restricted to the northwest of the Iberian Peninsula. Tetraploid var. *maritima* has a high probability of occurrence on the coast between Lariño and A Guarda in Spain (Figure 3B), corresponding to its current distribution (Figure 2). Tetraploid var. *sabularia* has a high likelihood of occurrence on the northern Portuguese coast, from Viana do Castelo to Figueira da Foz (Figure 3C), matching its current distribution, but also further south in areas where its presence was not detected.

The results for the putative contact zone revealed similar patterns (Figure 4). Suitable habitat for diploid var. *maritima* includes its current distribution range but extends further south along the Spanish coast (Figure 4A), overlapping the current distribution of tetraploid var. *maritima*. Suitable habitat for diploids also occurs around Costa Ártabra, but we did not detect natural populations in these coastal regions. The highest suitability for tetraploid var. *maritima* is found around the bay of Arousa and scattered also in the bays of Muros and Noia, corresponding to its current northern distribution (where it contacts with the diploid var. *maritima*; Figures 2, 4). Suitable habitats for tetraploid var. *maritima* also occur further south, particularly in southernmost regions of the Spanish coast (where it contacts with the tetraploid var. *sabularia*; Figures 2, 4) and in northernmost parts of the Portuguese coast (Figure 4B), where its presence was not detected (Figure 2). In the case of the tetraploid var. *sabularia*, suitable habitat extends beyond the observed distribution, to the north, where tetraploid var. *maritima* is currently found, and especially to the south (Figure 4C), where no *J. maritima* has been found (Figures 2, 4C).

**FIGURE 5:**
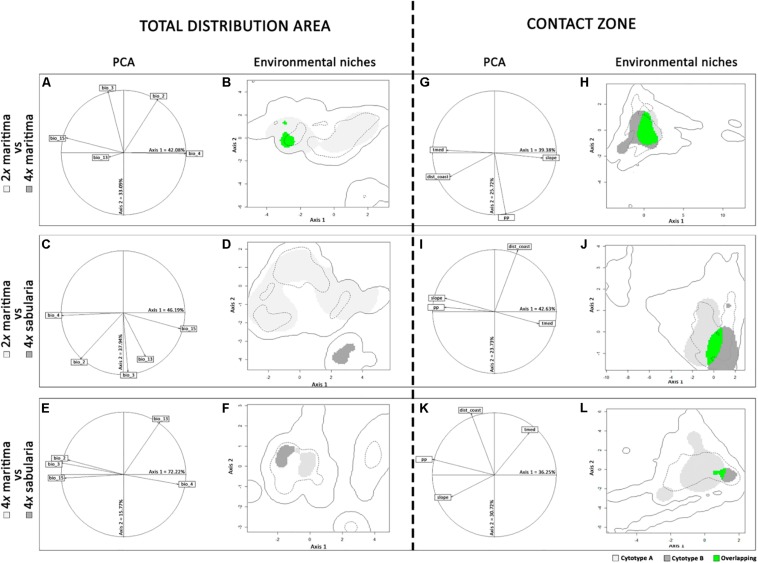
Ecological niche models for the varieties and cytotypes of *Jasione maritima* at: **(A–F)** the entire distribution area and **(G–L)** the Iberian Peninsula. **(A)**, **(C)**, **(E)**, **(G)**, **(I)**, and **(K)** represent the contribution of selected environmental variables [mean diurnal range (bio_2), isothermality (bio_3), temperature seasonality (bio_4), precipitation of the wettest month (bio_13) and precipitation seasonality (bio_15), slope, mean summer temperature (tmed), mean annual precipitation (pp) and distance to coast (dist)] to the first two axes of the principal component analyses (PCA), with the percentage of variance explained by each axis provided in each graphic; **(B)**, **(D)**, **(F)**, **(H)**, **(J)**, and **(K)** represent the environmental niche of diploid *J. maritima* var. *maritima* (2*x* maritima), tetraploid *J. maritima* var. *maritima* (4*x* maritima) and tetraploid *J. maritima* var. *sabularia* (4*x* sabularia), compared in pairs; colored areas represent the following: light gray – suitable habitats for variety/cytotype A, dark gray – suitable habitats for variety/cytotype B, and green – denotes the overlapping areas between cytotype A and B environmental niches; variety/cytotype A and B can be identified in the left of each panel; the continuous line corresponds to the whole climatic space, while the dashed line indicates the 75th percentile.

The first two axes of the PCAs explained a high percentage of environmental variance, namely over 75% for the entire distribution, and over 65% for the Iberian Peninsula (Figure 5; Supplementary Table 7). Across the entire species distribution range (Figures 5A–F), the environmental niche of tetraploid var. *maritima* falls completely within the environmental range of the diploid var. *maritima* (Figure 5B). Conversely, the amplitude of the environmental niche of diploids was much larger than that of the tetraploids, and only 6.9% of the diploid var. *maritima* environmental niche overlaps with the tetraploid var. *maritima* niche (dark gray area in Figure 5B). The environmental niche of tetraploid var. *sabularia* is distinguishable from diploid var. *maritima* mainly along axis 2, associated with isothermality and precipitation of the wettest month (Figures 5C,D and Supplementary Table 8), and from tetraploid var. *maritima* along axis 1, with strong associations with most of the selected variables (Figures 5E,F).

Analyses of the putative contact zone in the Iberian Peninsula (Figures 5G–L) show that the environmental ranges of the diploid and tetraploid cytotypes of var. *maritima* partially overlap; in particular, 68.0% of the diploid environmental niche overlaps with the tetraploid niche, and 53.7% for the reverse comparison (Figure 5H). The overlap between tetraploid var. *sabularia* and either cytotype of var. *maritima* is considerably lower (Figures 5J,L; Supplementary Table 7): 21.0% of the diploid var. *maritima* environmental niche and 3.6% of the tetraploid var. *maritima* niche overlaps with that of tetraploid var. *sabularia*; whereas 33.4 and 34.5% of the tetraploid var. *sabularia* environmental niche overlaps with diploid and tetraploid var. *maritima*, respectively. Also, the amplitude of the environmental niche of tetraploid var. *sabularia* was smaller, especially in comparison with that of tetraploid var. *maritima* (Figure 5L).

## Discussion

The niche shift hypothesis proposes that ecological divergence of polyploid lineages is a key process shaping the distribution of cytotypes in nature (Levin, 1975; Fowler and Levin, 1984). Here we provide detailed cytogeographical information for the two *J. maritima* varieties, diploid-tetraploid var. *maritima* and tetraploid var. *sabularia*, and explore the relationships between their environmental requirements and current distributions. Whereas tetraploid var. *sabularia* differed environmentally from diploid var. *maritima*, suggesting niche divergence, tetraploid var. *maritima* overlapped with the diploid, suggesting that other factors rather than niche divergence were involved in current distribution of tetraploid var. *maritima*. Additionally, tetraploid var. *maritima* currently occupies its entire predicted range, whereas diploids are restricted to the northern portion of their suitable environment only. Below we discuss these findings and its implications for the dynamics of diploid-polyploid complexes.

### Parapatric Distribution of *J. maritima* Varieties and Cytotypes

The current distributions of *J. maritima* varieties and cytotypes are clearly geographically segregated. Our chromosome counts revealed for the first time the presence of diploid individuals (2*n* = 2*x* = 12) in the northernmost localities of the Iberian Peninsula, which are confirmed by their lower genome size estimates (2.98 ± 0.07 pg) compared to tetraploids (6.06 ± 0.11 pg). Prior to this work, *J. maritima* on the Iberian Peninsula was considered to be only tetraploid (2*n* = 4*x* = 24; Lago Canzobre and Castroviejo, 1992; Rubido-Bará et al., 2010). Therefore, diploid var. *maritima* has a disjunct geographic distribution comprising the west coast of France and the northwest Iberian Peninsula. Although close affinities between the French and the Iberian populations have been pointed out (Parnell, 1987), the populations from these two regions differ significantly in their environmental variables. Such differences may reflect ecological divergence in allopatry and/or different evolutionary pathways. Further molecular studies are needed to unravel the relationships between French and Iberian populations.

Within the Iberian Peninsula, our survey revealed that the cytotypes and varieties are distributed parapatrically, with diploids observed in the north and tetraploids occupying more southern locations over an area that is larger than the one occupied by the diploids in this region. Two parapatric contact zones were observed, one between diploids and tetraploids of var. *maritima* in Lariño, within which populations of each cytotype are separated by a minimum of 8 kms, and another between tetraploids of var. *maritima* and *sabularia* at the Spanish-Portuguese border (Minho river separating var. *maritima* at north and var. *sabularia* at south). Spatial segregation between cytotypes has been observed in several other polyploid complexes (e.g., Husband and Schemske, 1998; Balao et al., 2009; Kolář et al., 2009; Sonnleitner et al., 2010; Castro et al., 2012, 2018; Casazza et al., 2016; Wefferling et al., 2017). Spatial segregation reduces inter-cytotype interactions and constitutes a physical barrier that prevents gene flow between cytotypes (Segraves and Thompson, 1999; Husband and Schemske, 2000; Baack, 2005; Nuismer and Cunningham, 2005) and weakens frequency-dependent selection on minority cytotypes (Levin, 1975). Consequently, it is widely viewed as a significant contributor to escape direct competition with diploid counterparts (Levin, 2002; Li et al., 2004; Baack and Stanton, 2005). Parapatric distribution may reflect competitive interactions or frequency dependent mating disadvantage, which prevent one cytotype from invading another cytotype area (see below). Reproductive isolation among *J. maritima* cytotypes and varieties mediated by current spatial segregation might promote evolutionary divergence, especially if the cytotypes and varieties are subjected to different selective pressures across the latitudinal range occupied by the species. In the long term, this could lead to the formation of distinct taxa (Otto and Whitton, 2000; Soltis et al., 2010).

### Environmental Niche Shifts

The north-south sequential linear distribution of *J. maritima* cytotypes and varieties fits the coastline of a transitional biogeographic unit between the Mediterranean and Atlantic regions (the Galicia and North Portugal biogeographic sector; Rivas-Martínez et al., 2017) and a limit to the distribution of several species. The non-overlapping ranges support a process of differentiation along an environmental gradient with each variety and cytotype occupying a subset of environmental conditions along a thermal and precipitation gradient representing an increase of Mediterranean conditions from north to south. In this context, the tetraploids of the two *J. maritima* varieties revealed different degrees of environmental divergence in comparison with their diploid counterpart. Our analyses indicate that the environmental niche of tetraploid var. *sabularia* is distinguishable from both diploid and tetraploid var. *maritima* cytotypes. In particular, the tetraploid var. *sabularia* colonized areas facing S-SW, which are hotter and slightly drier environments than those colonized by the var. *maritima*. These environmental differences result in little environmental niche overlap between the tetraploid var. *sabularia* and both cytotypes of var. *maritima*, suggesting that tetraploid var. *sabularia* has different tolerances to water availability and temperature, which allowed them to colonize areas beyond those suitable for the diploids. The capacity of polyploids to grow in environments that differ from their progenitor(s) allows polyploid lineages to expand to new areas where their lower ploidy parental(s) are absent (Maherali et al., 2009; Laere et al., 2011; Manzaneda et al., 2012, 2015; Hao et al., 2013) and, thus, avoid minority cytotype exclusion (Levin, 1975; Fowler and Levin, 1984; Felber, 1991). While we do not know whether the ecological differences arose in association with genome duplication or after separation from diploid var. *maritima*, our results are consistent with the importance of niche differentiation for successful establishment of polyploids. Further studies assessing physiological responses under contrasting water availabilities and temperatures are needed to experimentally assess their impacts on cytotype fitness.

In contrast to var. *sabularia*, the environmental niche of tetraploid var. *maritima* overlaps with the environmental niche of diploids. Additionally, the geographical distributions and the high-resolution analyses of habitat suitability at the putative contact zone shows that diploids seem to be confined to the northern suitable places, while the tetraploids occupy most of their suitable areas. Thus, although some degree of niche expansion is detected, there is poor evidence that whole genome duplications that led to the formation of the tetraploid var. *maritima* and/or posterior processes of divergence resulted in a shift in environmental niche in comparison with the diploid, and other factors rather than niche divergence must have been involved in the successful spread of tetraploid var. *maritima*. Interestingly, tetraploids of var. *maritima* invest more in belowground biomass than diploids, a trait that could have provided an advantage to colonize southern locations more severely affected by drought in comparison with northern locations where diploids occur (Castro, 2018). In the closely related diploid-tetraploid *J. montana*, environmental niche analyses also revealed that the tetraploid niche was nested within the diploid niche breadth, and environmental sorting was proposed as a determinant for their successful establishment (Castro et al., 2019). Although the occurrence of niche shifts in association with polyploidization has been described in some diploid-polyploid systems (e.g., Hao et al., 2013; Thompson et al., 2014; Visger et al., 2016; Muñoz-Pajares et al., 2018), there is also evidence in the literature supporting the idea that polyploids and their diploid progenitors occupy similar environmental conditions (e.g., Godsoe et al., 2013; Castro et al., 2019; reviewed in Glennon et al., 2014; Spoelhof et al., 2017). This supports the hypothesis that the success of the tetraploids might be context-dependent and species – or even genotype – specific. Thus, niche divergence could have been key in certain systems while in other processes, alone or combined, could have contributed for the current distribution patterns of the tetraploid var. *maritima*.

### Current Distribution Patterns at the Zones of Sympatry

While in northernmost areas environmental variables seem to explain the current distribution range of tetraploid var. *maritima*, other factors may prevent diploids from thriving within the tetraploid’s environmental niche. Increased competitive ability of polyploids may have allowed them to overcome frequency-dependent selection (Levin, 1975; Fowler and Levin, 1984; Rodríguez, 1996) and exclude diploids from invading these sites. Evidence for strong polyploid competitive ability in other species is conflicting (e.g., Maceira et al., 1993; Collins et al., 2011; Thompson et al., 2015). For example, tetraploid *Dactylis glomerata* is highly competitive compared to diploids (Maceira et al., 1993), whereas diploid and tetraploid *C. angustifolium* (Thompson et al., 2015) have similar competitive abilities. Relative competitive abilities of diploid and tetraploid *Centaurea stoebe* vary across the distribution range (Collins et al., 2011). In *J. maritima*, similar competitive abilities may promote a stable contact zone, whereas different competitive abilities are expected to generate a moving contact zone (e.g., Maceira et al., 1993), toward the south if diploids are more competitive, or to the north if tetraploids are stronger competitors, in any case expanding cytotype area until the environmental limit of the strongest competitor is reached. Similarly, in the southernmost zone of sympatry between tetraploid varieties, competitive ability may also have played an important role in the dynamics of the putative contact zone, as models predict that tetraploid var. *maritima* could occur further south in Portuguese coast and tetraploid var. *sabularia* could occur further north in Spanish territory. Therefore, competition experiments are needed to evaluate the role of competitive ability in shaping current cytotype distribution patterns in *J. maritima*.

Alternatively, reduced dispersal ability could limit the expansion of diploids to locations where tetraploid var. *maritima* grows. The seeds of *Jasione* species have no obvious dispersal mechanism. Like the seeds of *J. montana*, those of *J. maritima* are completely smooth; they are not adapted to water dispersal, as they immediately sink (Praeger, 1913; M. Castro, personal observations), nor to endozoochory, as it detrimentally affects germination levels (Peco et al., 2006). Previous experiments have also shown that the maximum seed dispersal distance seldom exceeds 1.4 m in *J. montana* (Parnell, 1985). Seed dispersal in *J. maritima* seems also to be hindered by the coast line characteristics, namely the restricted linear shape of dune habitat; the existence of spatial discontinuity in habitat associated with the occurrence of rocky outcrops reaching the coast and interrupting the dune system; the existence of deep gulfs, especially in the NW coast of Spain; and the presence of large end sections of rivers with significant discharge along the year (e.g., Minho river, or the wide Aveiro coastal lagoon-estuarine system at the current southern limit of var. *sabularia*). Nevertheless, even if diploids were capable of dispersing to southern locations, they may not find a suitable site to establish or may experience strong frequency dependent selection within tetraploid populations (Levin, 1975; Husband, 2000), particularly being a self-incompatible plant (Siopa et al., 2020). Occasional migration from the diploid area into the tetraploid range may allow some rates of gene flow (e.g., through triploid bridge) that could attenuate divergence in environmental requirements between cytotypes of var. *maritima.* In the absence of other fitness advantages, diploids would be eliminated from tetraploid populations and the current parapatric distribution maintained.

### Hypothesis About the Origins of the Two Tetraploids and Implications for Biodiversity

The evolutionary history of *Jasione* is still largely unknown. The few available phylogenetic studies suggest a recent origin of the species within the genus, linked with Pleistocene glaciation (Sales et al., 2004; Pérez-Espona et al., 2005). Consequently, we have limited evidence on which to build hypotheses regarding the origin of the tetraploids. *J. maritima* is closely related to the widespread *J. montana* (Bokhari and Sales, 2001; Pérez-Espona et al., 2005), and French and Spanish diploid populations have been considered a coastal sand-dune form of the inland *J. montana* (Parnell, 1980, 1982), although other authors have stressed the differences between *J. maritima* and some coastal forms of *J. montana* (Dupont, 2015). The data available for the Iberian Peninsula points that, on one hand, the diploids and tetraploids of var. *maritima* are very difficult to differentiate morphologically (Sales and Hedge, 2001), and were shown here to share similar environmental niches, two features usually associated with autopolyploid complexes (e.g., Glennon et al., 2012, 2014; Godsoe et al., 2013). On the other hand, the tetraploid var. *sabularia*, although closely resembling var. *maritima* in morphological and anatomical characters (Bokhari and Sales, 2001), exhibits some subtle morphological differences, such as sparser indumentum and different leaf shape that have led some taxonomists to treat it separately (e.g., as *J. lusitanica* in Flora Europaea; Tutin et al., 1976). In addition, genetic analyses using AFLPs separated var. *sabularia* from var. *maritima* and the closely related *J. montana* (Pérez-Espona et al., 2005). Unfortunately, based on the sampling locations, those studies likely only included diploid var. *maritima*. This previous research, together with the detection of niche shifts in comparison with the diploids, could suggest a separate divergence from the diploid parental or an allopolyploid origin of *J. maritima* var. *sabularia*. Allopolyploids often show either intermediate morphological features between putative parental diploids or transgressive phenotypes (Soltis et al., 2007), and this could the case of var. *sabularia*. Still, it is important to note that the different possibilities for the origins of the tetraploid varieties complicate the interpretation of niche divergence relative to the diploid var. *maritima* that span such great ecological space.

The origins of *J. maritima* seem compatible with a diversification model by which glacial refugia in the Iberian coast would have acted as triggers of speciation (e.g., González-Sampériz et al., 2010; Fernández-Mazuecos and Vargas, 2013). During this period, an ecotype of *J. montana* adapted to maritime sand-dunes could have survived in these coastal areas, and with the temperature fluctuation given rise to tetraploid cytotypes (once or multiple times); the tetraploids could have arisen through autopolyploidization or after hybridization with other taxa (allopolyploidization). At the current state of knowledge, we can only propose hypotheses, and phylogenetic and phylogeographic studies are necessary to test them and shed light on the timing and frequency of whole genome duplications in the genus. That said, our analyses of cytotype distribution and environmental niche variation within *J. maritima* polyploidy complex set a framework for appropriate hypothesis testing, and the detected ecological differences can contribute to understanding the internal biodiversity in the complex. In *J. maritima*, populations differing in ploidy can exhibit niche differences, as in tetraploid var. *sabularia* vs. diploid var. *maritima*, or differences in niche occupancy and geographical space, for which differences in competitive abilities could be invoked (Laport et al., 2013), as in tetraploid var. *maritima* vs. diploid var. *maritima*. Ultimately, each cytotype and variety probably has unique responses to the abiotic environment and to biotic interactions (Segraves and Anneberg, 2016), having clearly separate ranges that support the idea that they work as different functional units of biodiversity (Ramsey and Ramsey, 2014; Laport and Ng, 2017). Despite traditional reluctance to incorporate intraspecific ploidy differences into taxonomic decisions, particularly when no striking morphological differences are exhibited, as is frequent in autopolyploids (Soltis et al., 2007), our results support the recognition of diverse biodiversity units both between cytotypes and within the tetraploid cytotype in the *J. maritima* complex. Therefore, the study of differences and similarities in ecological envelopes among ploidy units is a valuable tool and should inform taxonomic and management decisions, particularly when the response to environmental factors (e.g., climate change) could be specific of each of the different units.

## Conclusion

In this study, we show that *J. maritima* has two parapatric contact zones in the Iberian Peninsula along a linear coastal distribution, one between diploid and tetraploid populations of the same variety (var. *maritima*), and the other between tetraploids from two different varieties (var. *maritima* and var. *sabularia*). The two tetraploids have different degrees of environmental divergence in comparison with the diploid. Whereas changes in environmental requirements may have allowed tetraploid var. *sabularia* to spread in habitats not favorable to its diploid progenitor, other factors such as ploidy interactions and competitive ability, have influenced the spread of tetraploid var. *maritima*. More studies, such as phylogenetic and phylogeographic reconstructions, and reciprocal transplants, competition and drought experiments, are needed to test these hypotheses. Differences in ecological tolerances can suggest the presence of different functional units of biodiversity within polyploid groups, allowing the study of factors related to post-polyploidization divergence.

## Data Availability Statement

The datasets generated for this study are available on request to the corresponding author.

## Author Contributions

MC, JL, MS, and SC designed the experiment and conducted field collections. MC conducted laboratory analysis. MC, AF, and SC performed the niche modeling analyses. All authors participated in scientific discussion and approved the submitted version.

## Conflict of Interest

The authors declare that the research was conducted in the absence of any commercial or financial relationships that could be construed as a potential conflict of interest.

## Footnotes

1 http://GBIF.org

2 http://www.worldclim.org

3 http://srtm.csi.cgiar.org

4 http://www.jennessent.com/arcview/tpi.htm

5 http://www.opengis.uab.es/wms/iberia/index.htm

6 http://datos.gob.es/es; Atlas Digital do Ambiente

## Supplementary Material

FIGURE S1 Principal component analyses: **(A)** for the entire geographic distribution of *J. maritima* (in Portugal, Spain and France), using 19 bioclimatic variables (Bio1-Bio19) plus elevation extracted for all species occurrences from the Worldclim database at a resolution of 1 km; **(B)** for the contact zone (in the northwest Iberian Peninsula), using the following set of variables at 100 m resolution – elevation (ele); aspect, slope, slope range (slprng) and topographic position index (tpi); summer mean temperature (tmed) and mean annual precipitation (pp); lithology (lito); and distance to coast (dist_coast). Variance explained by each axis is also provided.

TABLE S1 Geographic information of the *Jasione maritima* populations sampled in this study for flow cytometric analyses. For each population, an ID code, locality name, herbarium code at SANT, geographical coordinates (angular), sample size (N, number of individuals analyzed) and estimated DNA ploidy level are given. Ploidy levels: diploids (2*x*) and tetraploids (4*x*). The two ID codes marked with an asterisk (^∗^) denote populations where seeds were collected for chromosome counts.

TABLE S2 Environmental variables characterization in *Jasione maritima* considering the total distribution area. For each environmental variable mean and standard error of the mean (se) per cytotype are presented. *F* and *P* values are also presented. Different letter corresponds to statistically differences (*P* < 0.05) between the groups for a given environmental variable. Shades highlight differences between cytotypes/varieties. Bold highlight variables used in niche modeling.

TABLE S3 Environmental variables characterization in *Jasione maritima* in the contact zone (northwest of Iberian Peninsula) area. For each variable mean and standard error of the mean (se) are presented. *F* and *P* values are also presented. Different letter corresponds to statistically differences (*P* < 0.05) between the group within the same environmental variable. Shades highlight differences between cytotypes/varieties. Bold highlight variables used in niche modeling.

TABLE S4 Variable contribution in Principal Component Analyses using all variables (Figure 1) at total distribution area and contact zone analyses.

TABLE S5 *Jasione maritima* models’ evaluation. For diploid individuals of *J. maritima* var. *maritima* (2*x* var. *maritima*), tetraploid individuals of *J. maritima* var. *maritima* (4*x* var. *maritima*) and tetraploid individuals of *J. maritima* var. *sabularia* (4*x* var. *sabularia*) mean and standard error of AUC values of the models and of omission rates were presented for each cytotype in the different approaches. The value of omission rate of the final model for each cytotype/approach were also presented.

TABLE S6 Genome size estimates in *Jasione maritima*. In each population, DNA ploidy estimates and mean, standard deviation of the mean (SD), coefficient of variation (CV, in %), minimum (Min) and maximum (Max) values of holoploid genome size (2C, in pg) are given. Number of individuals analyzed for genome size in each population (*n*) and mean monoploid genome size (1C*x*, in pg) are also provided. Ploidy levels: diploids (2*x*) and tetraploids (4*x*).

TABLE S7 *Jasione maritima* environmental niches overlapping comparing the two varieties and two cytotypes in pairs (diploid niche of *J. maritima* var. *maritima* – 2*x* var. *maritima*, tetraploid niche of *J. maritima* var. *maritima* – 4*x* var. *maritima* and tetraploid niche of *J. maritima* var. *sabularia* – 4*x* var. *sabularia*). The percentage of variance explained the two-principal axis, the total of variance explained, percentage of environmental niche overlapping of cytotype A niche in cytotype B niche (**A → B**) and cytotype B niche in cytotype A niche (**B → A**) were present for each comparison in the two approaches.

TABLE S8 Variable contribution in Principal Component Analyses using selected variables using in the models (Figure 5) at total distribution area and contact zone analyses.
