# Corrigendum: Different Patterns of Ecological Divergence Between Two Tetraploids and Their Diploid Counterpart in a Parapatric Linear Coastal Distribution Polyploid Complex

**DOI:** 10.3389/fpls.2020.00676

**Published:** 2020-06-02

**Authors:** Mariana Castro, João Loureiro, Albano Figueiredo, Miguel Serrano, Brian C. Husband, Sílvia Castro

**Affiliations:** ^1^Centre for Functional Ecology, Department of Life Sciences, University of Coimbra, Coimbra, Portugal; ^2^Centre for Studies in Geography and Spatial Planning, Department of Geography and Tourism, Faculty of Arts and Humanities, University of Coimbra, Coimbra, Portugal; ^3^Department of Botany, Faculty of Pharmacy, University of Santiago de Compostela, Santiago de Compostela, Spain; ^4^Department of Integrative Biology, University of Guelph, Guelph, ON, Canada

**Keywords:** parapatric distribution, cryptic diversity, diploids, *Jasione maritima*, niche modeling, tetraploids

In the original article, we neglected to include the grant number “UID/BIA/04004/2020” for the Portuguese Foundation for Science and Technology (FCT). The corrected **Funding** statement appears below.

## Funding

This research was supported by POPH/FSE funds by the Portuguese Foundation for Science and Technology (FCT) with a doctoral grant to MC (SFRH/BD/89910/2012), a starting grant and exploratory project to SC (IF/01267/2013) and the project UID/BIA/04004/2020, by Xunta de Galicia with the grant to MS (ED431B 2018/36), and by Project RENATURE financed by the “Programa Operacional Regional do Centro 2014-2020 (Centro2020) – CENTRO-01-0145-FEDER-000007”.

The authors apologize for this error and state that this does not change the scientific conclusions of the article in any way. The original article has been updated.

