# Supplementary figures and images for: Different Patterns of Ecological Divergence Between Two Tetraploids and Their Diploid Counterpart in a Parapatric Linear Coastal Distribution Polyploid Complex
Source: Front Plant Sci. 2020 Mar 19;11:315. doi: 10.3389/fpls.2020.00315 (PMC7098452; doi:10.3389/fpls.2020.00315)

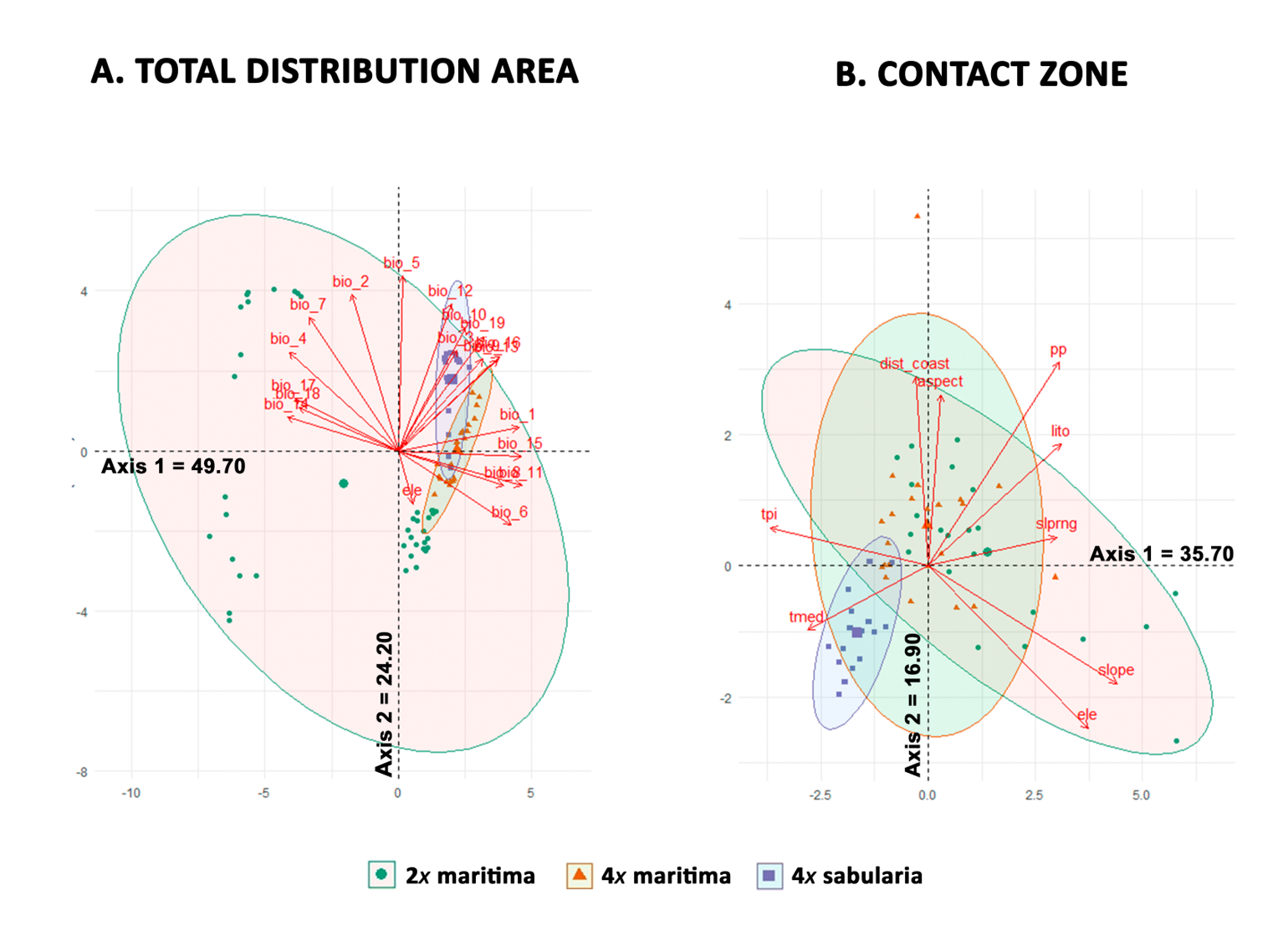

Supplement: FIGURE S1 — Principal component analyses: (A) for the entire geographic distribution of J. maritima (in Portugal, Spain and France), using 19 bioclimatic variables (Bio1-Bio19) plus elevation extracted for all species occurrences from the Worldclim database at a resolution of 1 km; (B) for the contact zone (in the northwest Iberian Peninsula), using the following set of variables at 100 m resolution – elevation (ele); aspect, slope, slope range (slprng) and topographic position index (tpi); summer mean temperature (tmed) and mean annual precipitation (pp); lithology (lito); and distance to coast (dist_coast). Variance explained by each axis is also provided. [file Image_1.TIF]
