# Supplementary material for: Different Patterns of Ecological Divergence Between Two Tetraploids and Their Diploid Counterpart in a Parapatric Linear Coastal Distribution Polyploid Complex
Source: Front Plant Sci. 2020 Mar 19;11:315. doi: 10.3389/fpls.2020.00315 (PMC7098452; doi:10.3389/fpls.2020.00315)
Supplement: TABLE S1 — Geographic information of the Jasione maritima populations sampled in this study for flow cytometric analyses. For each population, an ID code, locality name, herbarium code at SANT, geographical coordinates (angular), sample size (N, number of individuals analyzed) and estimated DNA ploidy level are given. Ploidy levels: diploids (2x) and tetraploids (4x). The two ID codes marked with an asterisk (∗) denote populations where seeds were collected for chromosome counts. [file Table_1.docx]

**Table S1.** Geographic information of the *Jasione maritima* populations sampled in this study for flow cytometric analyses. For each population, an ID code, locality name, herbarium code at SANT, geographical coordinates (angular), sample size (N, number of individuals analyzed) and estimated DNA ploidy level are given. Ploidy levels: diploids (2*x*) and tetraploids (4*x*). The two ID codes marked with an asterisk (*) denote populations where seeds were collected for chromosome counts.

| **ID code** | **Population** |  | **Geographical coordinates** | | **N** | **Ploidy** |  |
| --- | --- | --- | --- | --- | --- | --- | --- |
|  |  |  | **Latitude** | **Longitude** |  |  |  |
| ***J. maritima* var. *maritima*** | | | | | | | |
| MS001 | Lage, Soesto, La Coruña, Spain |  | 43.21240 | -9.02343 | 30 | 2*x* |  |
| MS002 | Pedrosa beach, Mourín, La Coruña, Spain |  | 43.15818 | -9.19126 | 30 | 2*x* |  |
| MS003* | Lourido, La Coruña, Spain |  | 43.08677 | -9.22109 | 30 | 2*x* |  |
| MS046 | Monte Branco, Pontecesso, La Coruña, Spain |  | 43.23429 | -8.93088 | 37 | 2*x* |  |
| SC073 | Lariño, La Coruña, Spain |  | 42.77103 | -9.12227 | 30 | 2*x* |  |
| SC074 | Lira, La Coruña, Spain |  | 42.80479 | -9.12781 | 32 | 2*x* |  |
| SC076 | Fisterra, Rostro beach, La Coruña, Spain |  | 42.91861 | -9.26416 | 30 | 2*x* |  |
| SC077 | Fisterra, Afora beach, La Coruña, Spain |  | 42.90851 | -9.27328 | 33 | 2*x* |  |
| SC150 | Nemiña, Talón, La Coruña, Spain |  | 43.00983 | -9.26141 | 4 | 2*x* |  |
| SC242 | Boaño, La Coruña, Spain |  | 43.19168 | -9.04252 | 12 | 2*x* |  |
| SC243 | Balarés, Pontecesso, La Coruña, Spain |  | 43.24197 | -8.94148 | 12 | 2*x* |  |
| SC244 | Casas da Hermida, La Coruña, Spain |  | 43.26401 | -8.9512 | 7 | 2*x* |  |
| MC369 | Couso, La Coruña, Spain |  | 42.52006 | -9.03848 | 44 | 4*x* |  |
| SC070 | Testal, Taramancos, La Coruña, Spain |  | 42.79078 | -8.91341 | 30 | 4*x* |  |
| SC071 | Esteiro, La Coruña, Spain |  | 42.79029 | -8.97947 | 49 | 4*x* |  |
| SC072 | Ventim, Abelheira, La Coruña, Spain |  | 42.79917 | -9.02685 | 35 | 4*x* |  |
| SC078 | Cans, La Coruña, Spain |  | 42.74260 | -8.96409 | 29 | 4*x* |  |
| SC079 | Tarela, La Coruña, Spain |  | 42.67273 | -9.03290 | 30 | 4*x* |  |
| SC080 | Basoña, La Coruña, Spain |  | 42.61898 | -9.05401 | 35 | 4*x* |  |
| SC083 | Caiños, La Coruña, Spain |  | 42.58534 | -8.94885 | 30 | 4*x* |  |
| SC084 | Fonte de Mouro, La Coruña, Spain |  | 42.61228 | -8.87213 | 30 | 4*x* |  |
| SC085 | Con Cerrado, Illa Arousa, Pontevedra, Spain |  | 42.53166 | -8.86943 | 30 | 4*x* |  |
| SC113 | A Lanzada, O Grove, Spain |  | 42.44249 | -8.87156 | 27 | 4*x* |  |
| SC114 | Barbeito, Pontevedra, Spain |  | 42.39955 | -8.85051 | 30 | 4*x* |  |
| SC116* | Liméns, Pontevedra, Spain |  | 42.26023 | -8.8137 | 35 | 4*x* |  |
| SC117 | Baiona, Pontevedra, Spain |  | 42.11335 | -8.82828 | 35 | 4*x* |  |
| SC118 | A Praia, Pontevedra, Spain |  | 41.87318 | -8.86698 | 29 | 4*x* |  |
| ***J. maritima* var. *sabularia*** | | | | | | | |
| MC215 | Torreira, Aveiro, Portugal |  | 40.75708 | -8.71291 | 36 | 4*x* |  |
| MC216 | Esmoriz, Portugal |  | 40.95983 | -8.65245 | 3 | 4*x* |  |
| MC217 | Sisto, Esmoriz, Portugal |  | 40.98698 | -8.64463 | 32 | 4*x* |  |
| MC218 | Marinha, Vila Nova de Gaia, Portugal |  | 41.09783 | -8.65881 | 30 | 4*x* |  |
| MC219 | Aguçadeira, Porto, Portugal |  | 41.44315 | -8.77734 | 30 | 4*x* |  |
| MC220 | Anha, Viana do Castelo, Portugal |  | 41.66749 | -8.82249 | 30 | 4*x* |  |
| MC238 | Furadouro, Aveiro, Portugal |  | 40.87816 | -8.67341 | 10 | 4*x* |  |
| SC028 | Angeiras, Porto, Portugal |  | 41.26942 | -8.72622 | 30 | 4*x* |  |
