# Supplementary material for: Different Patterns of Ecological Divergence Between Two Tetraploids and Their Diploid Counterpart in a Parapatric Linear Coastal Distribution Polyploid Complex
Source: Front Plant Sci. 2020 Mar 19;11:315. doi: 10.3389/fpls.2020.00315 (PMC7098452; doi:10.3389/fpls.2020.00315)
Supplement: TABLE S2 — Environmental variables characterization in Jasione maritima considering the total distribution area. For each environmental variable mean and standard error of the mean (se) per cytotype are presented. F and P values are also presented. Different letter corresponds to statistically differences (P < 0.05) between the groups for a given environmental variable. Shades highlight differences between cytotypes/varieties. Bold highlight variables used in niche modeling. [file Table_2.docx]

**Table S2.** Environmental variables characterization in *Jasione maritima* considering the total distribution area. For each environmental variable mean and standard error of the mean (se) per cytotype are presented. Statistically *F* and *P* values are also presented. Different letter corresponds to statistically differences (*P* < 0.05) between the groups for a given environmental variable. Shades highlight differences between cytotypes/varieties. Bold highlight variables used in niche modelling.

| **Variables** | **CODE** | **2*x* var. *maritima*** | **4*x* var. *maritima*** | **4*x* var. *sabularia*** | **ANOVA**  *F_2,76_* and *P* values | |
| --- | --- | --- | --- | --- | --- | --- |
|  |  | mean ± se, n = 40 | mean ± se, n = 21 | mean ± se, n = 18 |  |  |
| Annual mean temperature | Bio1 | 137.15 ± 1.84^a^ | 148.29 ± 0.27^b^ | 146.06 ± 0.27^b^ | 14.68 | <0.001 |
| **Mean diurnal range** | Bio2 | 68.15 ± 1.83^a^ | 60.24 ± 0.56^b^ | 80.67 ± 0.79^c^ | 27.89 | <0.001 |
| **Isothermality** | Bio3 | 38.90 ± 0.23^a^ | 38.67 ± 0.21^a^ | 44.83 ± 0.15 ^b^ | 179.16 | <0.001 |
| **Temperature seasonality** | Bio4 | 3766.20 ± 114.67^a^ | 3375.86 ± 17.91^b^ | 3520.44 ± 27.81^ab^ | 4.13 | 0.020 |
| Maximum temperature of warmest month | Bio5 | 230.68 ± 1.96^a^ | 231.90 ± 0.37^ab^ | 237.89 ± 1.17^b^ | 3.90 | 0.025 |
| Minimum temperature of coldest month | Bio6 | 57.73 ± 3.54^a^ | 78.57 ± 0.59^b^ | 60.11 ± 0.63^a^ | 12.08 | <0.001 |
| Temperature annual range | Bio7 | 172.95 ± 4.51^a^ | 153.33 ± 0.82^b^ | 177.78 ± 1.64^a^ | 8.28 | <0.001 |
| Mean temperature of wettest quarter | Bio8 | 101.15 ± 3.11^a^ | 113.76 ± 0.46^b^ | 104.39 ± 1.11^ab^ | 5.41 | 0.006 |
| Mean temperature of driest quarter | Bio9 | 181.53 ± 1.31^a^ | 190.29 ± 0.40^b^ | 188.61 ± 0.43^b^ | 17.44 | <0.001 |
| Mean temperature of warmest quarter | Bio10 | 186.18 ± 1.31^a^ | 192.24 ± 0.33^b^ | 190.39 ± 0.44^b^ | 7.80 | <0.001 |
| Mean temperature of coldest quarter | Bio11 | 89.58 ± 3.14^a^ | 105.52 ± 0.36^b^ | 100.28 ± 0.30^b^ | 9.52 | <0.001 |
| Annual precipitation | Bio12 | 1007.50 ± 19.93^a^ | 1172.76 ± 17.80^b^ | 1158.67 ± 16.51^b^ | 22.70 | 1.9e-8 |
| **Precipitation of wettest month** | Bio13 | 133.63 ± 2.58^a^ | 164.76 ± 2.41^b^ | 159.11 ± 2.56^b^ | 42.23 | <0.001 |
| Precipitation of driest month | Bio14 | 38.15 ± 2.38^a^ | 25.76 ± 0.44^b^ | 13.67 ± 0.23^c^ | 33.08 | <0.001 |
| **Precipitation seasonality** | Bio15 | 34.68 ± 1.39^a^ | 46.24 ± 0.38^b^ | 51.61 ± 0.14^c^ | 51.22 | <0.001 |
| Precipitation of wettest quarter | Bio16 | 370.93 ± 6.62^a^ | 468.90 ± 7.36^b^ | 464.44 ± 6.24^b^ | 67.08 | <0.001 |
| Precipitation of driest quarter | Bio17 | 145.90 ± 7.53^a^ | 108.86 ± 0.98^b^ | 77.67 ± 1.07^c^ | 26.30 | <0.001 |
| Precipitation of warmest quarter | Bio18 | 170.23 ± 7.68^a^ | 132.19 ± 1.02^b^ | 95.89 ± 1.58^c^ | 29.38 | <0.001 |
| Precipitation of coldest quarter | Bio19 | 326.23 ± 5.74^a^ | 426.67 ± 9.41^b^ | 456.33 ± 7.98^c^ | 93.49 | <0.001 |
| Elevation | Alt | 19.28 ± 4.60 | 16.38 ± 4.77 | 8.00 ± 1.29 | 1.39 | 0.254 |
| Longitude | Long | -5.88 ± 0.58^a^ | -8.93 ± 0.02^b^ | -8.71 ± 0.01^b^ | 12.33 | <0.001 |
| Latitude | Lat | 44.30 ± 0.30^a^ | 42.52 ± 0.05^b^ | 41.16 ± 0.06^c^ | 34.86 | <0.001 |
