# Supplementary material for: Different Patterns of Ecological Divergence Between Two Tetraploids and Their Diploid Counterpart in a Parapatric Linear Coastal Distribution Polyploid Complex
Source: Front Plant Sci. 2020 Mar 19;11:315. doi: 10.3389/fpls.2020.00315 (PMC7098452; doi:10.3389/fpls.2020.00315)
Supplement: TABLE S3 — Environmental variables characterization in Jasione maritima in the contact zone (northwest of Iberian Peninsula) area. For each variable mean and standard error of the mean (se) are presented. F and P values are also presented. Different letter corresponds to statistically differences (P < 0.05) between the group within the same environmental variable. Shades highlight differences between cytotypes/varieties. Bold highlight variables used in niche modeling. [file Table_3.docx]

**Table S3.** Environmental variables characterization in *Jasione maritima* in the contact zone (northwest of Iberian Peninsula) area. For each variable mean and standard error of the mean (se) are presented. Statistically *F* and *P* values are also presented. Different letter corresponds to statistically differences (*P* < 0.05) between the group within the same environmental variable. Shades highlight differences between cytotypes/varieties. Bold highlight variables used in niche modelling.

| **Variables** | **CODE** | **2*x* var. maritima** | **4*x* var. maritima** | **4*x* var. sabularia** | **ANOVA**  *F_2,58_* and *P* values | |
| --- | --- | --- | --- | --- | --- | --- |
|  |  | mean ± se, n = 22 | mean ± se, n = 21 | mean ± se, n = 18 |  |  |
| Elevation | ele | 8.71 ± 1.57 | 5.22 ± 0.87 | 5.33 ± 0.40 | 2.99 | 0.059 |
| Aspect | aspect | 225.65 ± 22.87 (W) | 211.64 ± 20.90 (S/SW) | 198.67 ± 24.23 (S/SW) | 0.34 | 0.718 |
| **Distance from the sea** | dist | 182.55 ± 29.67 | 413.60 ± 199.02 | 139.80 ± 28.48 | 1.42 | 0.252 |
| Lithology | lito | 41.09 ± 1.76 ^a^ | 40.88 ± 1.55 ^a^ | 31.00 ± 0.00 ^b^ | 14.60 | <0.001 |
| **Mean annual precipitation** | pp | 1780.84 ± 22.94 ^a^ | 1798.31 ± 22.82 ^a^ | 1214.60 ± 29.08 ^b^ | 159.94 | <0.001 |
| **Slope** | slope | 4.20 ± 0.86 ^a^ | 1.54 ± 0.31 ^b^ | 0.89 ± 0.10 ^b^ | 9.03 | <0.001 |
| Incoming solar radiation in summer | slprng | 75.19 ± 4.45 ^a^ | 77.25 ± 4.76 ^a^ | 61.13 ± 1.39 ^b^ | 3.56 | 0.036 |
| **Mean summer temperature** | tmed | 13.84 ± 0.05 ^a^ | 14.42 ± 0.11 ^a^ | 14.63 ± 0.02 ^b^ | 32.52 | <0.001 |
| Topographic position index | tpi | -1.70 ± 0.66 ^a^ | -0.17 ± 0.27 ^b^ | -0.20 ± 0.25 ^b^ | 3.37 | 0.042 |
| Longitude | Long | -9.13 ± 0.02 ^a^ | -8.93 ± 0.02 ^b^ | -8.71 ± 0.01 ^c^ | 108.83 | <0.001 |
| Latitude | Lat | 43.03 ± 0.04 ^a^ | 42.52 ± 0.05 ^b^ | 41.16 ± 0.06 ^c^ | 363.70 | <0.001 |
