# Supplementary material for: Different Patterns of Ecological Divergence Between Two Tetraploids and Their Diploid Counterpart in a Parapatric Linear Coastal Distribution Polyploid Complex
Source: Front Plant Sci. 2020 Mar 19;11:315. doi: 10.3389/fpls.2020.00315 (PMC7098452; doi:10.3389/fpls.2020.00315)
Supplement: TABLE S4 — Variable contribution in Principal Component Analyses using all variables (Figure 1) at total distribution area and contact zone analyses. [file Table_4.docx]

**Table S4.** Variable contribution in Principal Component Analyses using all variables (Figure S1) at total distribution area and contact zone analyses.

| **Variables** | **All variables** | |
| --- | --- | --- |
|  |  |  |
|  | Axis 1 | Axis 2 |
| **Total distribution area (1km)** | | |
| bio_1 | 8.62 | 0.30 |
| bio_2 | -1.31 | 13.21 |
| bio_3 | 2.02 | 5.45 |
| bio_4 | -7.08 | 5.25 |
| bio_5 | 0.01 | 16.55 |
| bio_6 | 7.54 | -2.95 |
| bio_7 | -4.74 | 9.62 |
| bio_8 | 6.54 | -0.66 |
| bio_9 | 4.23 | 4.54 |
| bio_10 | 2.66 | 8.13 |
| bio_11 | 9.02 | -0.66 |
| bio_12 | 1.65 | 11.67 |
| bio_13 | 5.83 | 4.46 |
| bio_14 | -7.28 | 0.61 |
| bio_15 | 8.89 | -0.02 |
| bio_16 | 5.97 | 4.84 |
| bio_17 | -6.32 | 1.49 |
| bio_18 | -5.86 | 1.03 |
| bio_19 | 4.29 | 7.06 |
| ele | 0.13 | -1.50 |
| **Contact zone (100m)** | | |
| dist_coast | -0.09 | 21.33 |
| ele | 16.89 | -15.68 |
| aspect | 0.11 | 17.43 |
| lito | 11.56 | 8.84 |
| pp | 11.32 | 24.69 |
| slope | 23.42 | -8.23 |
| slprng | 10.86 | 0.49 |
| tmed | -9.48 | -2.45 |
| tpi | -16.27 | 0.87 |
