# Supplementary material for: Different Patterns of Ecological Divergence Between Two Tetraploids and Their Diploid Counterpart in a Parapatric Linear Coastal Distribution Polyploid Complex
Source: Front Plant Sci. 2020 Mar 19;11:315. doi: 10.3389/fpls.2020.00315 (PMC7098452; doi:10.3389/fpls.2020.00315)
Supplement: TABLE S5 — Jasione maritima models’ evaluation. For diploid individuals of J. maritima var. maritima (2x var. maritima), tetraploid individuals of J. maritima var. maritima (4x var. maritima) and tetraploid individuals of J. maritima var. sabularia (4x var. sabularia) mean and standard error of AUC values of the models and of omission rates were presented for each cytotype in the different approaches. The value of omission rate of the final model for each cytotype/approach were also presented. [file Table_5.docx]

**Table S5.** *Jasione maritima* models’ evaluation. For diploid individuals of *J. maritima* var. *maritima* (2*x* var. *maritima*), tetraploid individuals of *J. maritima* var. *maritima* (4*x* var. *maritima*) and tetraploid individuals of *J. maritima* var. *sabularia* (4*x* var. *sabularia*) mean and standard error of AUC values of the models and of omission rates were presented for each cytotype in the different approaches. The value of omission rate of the final model for each cytotype/approach were also presented.

| **Cytotypes** | **AUC values** | **Omission rates** | **Omission rates in the final model** |
| --- | --- | --- | --- |
|  | mean ± se |  |  |
| **Total distribution area (1km)** | | | |
| 2*x* var. *maritima* | 98.52 ± 0.11 | 0.08 ± 0.08 | 2.50 |
| 4*x* var. *maritima* | 94.93 ± 0.91 | 10.18 ± 1.83 | 4.80 |
| 4*x* var. *sabularia* | 98.67 ± 0.61 | 2.58 ± 1.22 | 0.00 |
| **Contact zone (100m)** | | | |
| 2*x* var. *maritima* | 99.37 ± 0.04 | 0.00 | 4.50 |
| 4*x* var. *maritima* | 98.30 ± 0.44 | 0.81 ± 0.81 | 3.81 |
| 4*x* var. *sabularia* | 98.60 ± 0.78 | 2.56 ± 1.55 | 0.00 |
