# Supplementary material for: Different Patterns of Ecological Divergence Between Two Tetraploids and Their Diploid Counterpart in a Parapatric Linear Coastal Distribution Polyploid Complex
Source: Front Plant Sci. 2020 Mar 19;11:315. doi: 10.3389/fpls.2020.00315 (PMC7098452; doi:10.3389/fpls.2020.00315)
Supplement: TABLE S6 — Genome size estimates in Jasione maritima. In each population, DNA ploidy estimates and mean, standard deviation of the mean (SD), coefficient of variation (CV, in %), minimum (Min) and maximum (Max) values of holoploid genome size (2C, in pg) are given. Number of individuals analyzed for genome size in each population (n) and mean monoploid genome size (1Cx, in pg) are also provided. Ploidy levels: diploids (2x) and tetraploids (4x). [file Table_6.docx]

**Table S6.** Genome size estimates in *Jasione maritima*. In each population, DNA ploidy estimates and mean, standard deviation of the mean (SD), coefficient of variation (CV, in %), minimum (Min) and maximum (Max) values of holoploid genome size (2C, in pg) are given. Number of individuals analyzed for genome size in each population (n) and mean monoploid genome size (1C*x*, in pg) are also provided. Ploidy levels: diploids (2*x*) and tetraploids (4*x*).

| ID code | Ploidy level |  | Homoploid genome size (2C) | | | | | |  | Monoploid Genome size (1C*x*) |
| --- | --- | --- | --- | --- | --- | --- | --- | --- | --- | --- |
|  |  |  | Mean | SD | CV (%) | Min | Max | N |  |  |
| *J. maritima* var*. maritima* | | | |  |  |  |  |  |  |  |
| MS003 | 2*x* |  | 2.93 | 0.01 | 0.4% | 2.92 | 2.94 | 3 |  | 0.25 |
| SC073 | 2*x* |  | 2.92 | 0.05 | 1.9% | 2.88 | 2.98 | 3 |  | 0.24 |
| SC077 | 2*x* |  | 2.89 | 0.04 | 1.3% | 2.84 | 2.91 | 3 |  | 0.25 |
| SC242 | 2*x* |  | 3.01 | 0.05 | 1.6% | 2.98 | 3.05 | 2 |  | 0.25 |
| SC243 | 2*x* |  | 3.02 | 0.04 | 1.5% | 2.91 | 3.06 | 10 |  | 0.25 |
| SC244 | 2*x* |  | 3.07 | 0.04 | 1.2% | 3.03 | 3.10 | 3 |  | 0.25 |
| MC369 | 4*x* |  | 6.11 | 0.16 | 2.6% | 6.00 | 6.29 | 3 |  | 0.25 |
| SC071 | 4*x* |  | 6.03 | 0.14 | 2.3% | 5.80 | 6.36 | 10 |  | 0.25 |
| SC072 | 4*x* |  | 6.11 | 0.09 | 1.5% | 5.97 | 6.22 | 10 |  | 0.25 |
| SC080 | 4*x* |  | 6.11 | 0.05 | 0.8% | 6.06 | 6.17 | 5 |  | 0.25 |
| SC116 | 4*x* |  | 6.03 | 0.08 | 1.4% | 5.93 | 6.16 | 5 |  | 0.25 |
| SC117 | 4*x* |  | 5.98 | 0.11 | 1.8% | 5.86 | 6.09 | 5 |  | 0.25 |
| *J. maritima var. sabularia* | | | |  |  |  |  |  |  |  |
| MC215 | 4*x* |  | 5.90 | 0.16 | 2.7% | 5.76 | 6.19 | 6 |  | 0.25 |
| MC217 | 4*x* |  | 5.86 | 0.09 | 1.6% | 5.79 | 5.92 | 2 |  | 0.24 |
| MC293 | 4*x* |  | 6.05 | 0.18 | 3.0% | 5.80 | 5.36 | 14 |  | 0.25 |
