# Supplementary material for: Different Patterns of Ecological Divergence Between Two Tetraploids and Their Diploid Counterpart in a Parapatric Linear Coastal Distribution Polyploid Complex
Source: Front Plant Sci. 2020 Mar 19;11:315. doi: 10.3389/fpls.2020.00315 (PMC7098452; doi:10.3389/fpls.2020.00315)
Supplement: TABLE S7 — Jasione maritima environmental niches overlapping comparing the two varieties and two cytotypes in pairs (diploid niche of J. maritima var. maritima – 2x var. maritima, tetraploid niche of J. maritima var. maritima – 4x var. maritima and tetraploid niche of J. maritima var. sabularia – 4x var. sabularia). The percentage of variance explained the two-principal axis, the total of variance explained, percentage of environmental niche overlapping of cytotype A niche in cytotype B niche (A → B) and cytotype B niche in cytotype A niche (B → A) were present for each comparison in the two approaches. [file Table_7.docx]

**Table S7.** *Jasione maritima* environmental niches overlapping comparing the two varieties and two cytotypes in pairs (diploid niche of *J. maritima* var. *maritima* – 2*x* var. *maritima*, tetraploid niche of *J. maritima* var. *maritima* – 4*x* var. *maritima* and tetraploid niche of *J. maritima* var. *sabularia* – 4*x* var. *sabularia*). The percentage of variance explained the two-principal axis, the total of variance explained, percentage of environmental niche overlapping of cytotype A niche in cytotype B niche (**A 🡪 B**) and cytotype B niche in cytotype A niche (**B 🡪 A**) were present for each comparison in the two approaches.

|  | **Cytotypes A** | **Cytotype B** | **PCA data** | | |  | **Overlapping** | |
| --- | --- | --- | --- | --- | --- | --- | --- | --- |
|  |  |  | **Axis 1** | **Axis 2** | **Total** |  | **A 🡪 B** | **B 🡪 A** |
| **Total distribution area (1 km)** | | |  |  |  |  |  |  |
|  | 2*x* var. *maritima* | 4*x* var. *maritima* | 33.09 | 42.08 | 75.17 |  | 6.92 | 100.00 |
|  |  | 4*x* var. *sabularia* | 46.19 | 37.94 | 84.13 |  | 0.00 | 0.00 |
|  | 4*x* var. *maritima* | 4*x* var. *sabularia* | 72.22 | 15.77 | 87.99 |  | 0.00 | 0.00 |
|  |  |  |  |  |  |  |  |  |
| **Contact zone (100 m)** | | |  |  |  |  |  |  |
|  | 2*x* var. *maritima* | 4*x* var. *maritima* | 25.72 | 39.38 | 65.10 |  | 68.04 | 53.73 |
|  |  | 4*x* var. *sabularia* | 42.63 | 23.73 | 66.36 |  | 21.06 | 33.44 |
|  | 4*x* var. *maritima* | 4*x* var. *sabularia* | 36.25 | 30.72 | 66.97 |  | 3.61 | 34.51 |
