# Supplementary material for: Different Patterns of Ecological Divergence Between Two Tetraploids and Their Diploid Counterpart in a Parapatric Linear Coastal Distribution Polyploid Complex
Source: Front Plant Sci. 2020 Mar 19;11:315. doi: 10.3389/fpls.2020.00315 (PMC7098452; doi:10.3389/fpls.2020.00315)
Supplement: TABLE S8 — Variable contribution in Principal Component Analyses using selected variables using in the models (Figure 5) at total distribution area and contact zone analyses. [file Table_8.docx]

**Table S8.** Variable contribution in Principal Component Analyses using selected variables using in the models (Figure 5) at total distribution area and contact zone analyses.

| **Variables** | | **Selected variables** | | | | | | | |  |
| --- | --- | --- | --- | --- | --- | --- | --- | --- | --- | --- |
|  |  | *2x_mar vs 4x_mar* | | | | *2x_mar vs 4x_sab* | | *4x_mar vs 4x_sab* | | |
|  |  | Axis 1 | | Axis 2 | | Axis 1 | Axis 2 | Axis 1 | Axis 2 |  |
| **Total distribution area (1km)** | | |  | |  | |  |  |  |  |
| bio_2 | | 146.43 | | 231.29 | | -177.71 | -191.66 | -92.50 | 24.55 |  |
| bio_3 | | -68.16 | | 264.72 | | 19.46 | -245.00 | -103.43 | 19.68 |  |
| bio_4 | | 266.95 | | 3.78 | | -256.87 | -10.66 | 91.06 | -15.90 |  |
| bio_13 | | -63.99 | | -18.47 | | 91.70 | -179.70 | 59.59 | 87.37 |  |
| bio_15 | | -247.86 | | 64.54 | | 236.98 | -65.62 | -98.48 | -5.56 |  |
| **Contact zone (100m)** |  | |  | |  | |  |  |  |  |
| dist_coast | | -172.53 | | -91.74 | | 72.50 | 192.94 | -79.73 | 210.35 |  |
| pp | | 43.47 | | -237.43 | | -157.03 | 14.25 | -211.07 | 53.31 |  |
| slope | | 184.12 | | -19.34 | | -154.80 | 42.35 | -147.52 | -74.45 |  |
| tmed | | -185.44 | | 10.49 | | 138.24 | -37.56 | 118.15 | 144.21 |  |
